# Efficacy and safety of veliparib plus chemotherapy for the treatment of lung cancer: A systematic review of clinical trials

**DOI:** 10.1371/journal.pone.0291044

**Published:** 2023-09-08

**Authors:** Amin Daei Sorkhabi, Asra Fazlollahi, Aila Sarkesh, Reza Aletaha, Hamidreza Feizi, Seyed Ehsan Mousavi, Seyed Aria Nejadghaderi, Mark J. M. Sullman, Ali-Asghar Kolahi, Saeid Safiri

**Affiliations:** 1 Student Research Committee, Tabriz University of Medical Sciences, Tabriz, Iran; 2 Hematology and Oncology Research Center, Tabriz University of Medical Sciences, Tabriz, Iran; 3 Neurosciences Research Center, Aging Research Institute, Tabriz University of Medical Sciences, Tabriz, Iran; 4 Tuberculosis and Lung Diseases Research Center, Tabriz University of Medical Sciences, Tabriz, Iran; 5 Systematic Review and Meta-analysis Expert Group (SRMEG), Universal Scientific Education and Research Network (USERN), Tehran, Iran; 6 Department of Life and Health Sciences, University of Nicosia, Nicosia, Cyprus; 7 Department of Social Sciences, University of Nicosia, Nicosia, Cyprus; 8 Social Determinants of Health Research Center, Shahid Beheshti University of Medical Sciences, Tehran, Iran; 9 Clinical Research Development Unit of Tabriz Valiasr Hospital, Tabriz University of Medical Sciences, Tabriz, Iran; 10 Social Determinants of Health Research Center, Department of Community Medicine, Faculty of Medicine, Tabriz University of Medical Sciences, Tabriz, Iran; BRAC University, BANGLADESH

## Abstract

**Background:**

As a poly-ADP ribose polymerase (PARP) inhibitor, veliparib has been identified as a potential therapeutic agent for lung cancer. The present study aimed to conduct a systematic review of clinical trials investigating the efficacy and safety of veliparib for treating lung cancer.

**Methods:**

PubMed, Scopus, the Web of Science, and Google Scholar were systematically searched up to October 30, 2022. Only randomized controlled trials (RCTs) evaluating the efficacy or safety of veliparib in the treatment of lung cancer patients were included. Studies were excluded if they were not RCTs, enrolled healthy participants or patients with conditions other than lung cancer, or investigated therapeutic approaches other than veliparib. The Cochrane risk-of-bias tool was used for quality assessment.

**Results:**

The seven RCTs (n = 2188) showed that patients treated with a combination of veliparib and chemotherapy had a significantly higher risk of adverse events, when compared to the control arm. There was no statistically significant difference in overall survival (OS) between those treated with veliparib plus chemotherapy and those receiving the standard therapies. Only two trials demonstrated an improvement in progression-free survival (PFS), and only one study found an increase in objective response rate (ORR). Furthermore, adding veliparib to standard chemotherapy showed no benefit in extending the duration of response (DoR) in any of the studies.

**Conclusions:**

Only a small number of studies have found veliparib to be effective, in terms of improved OS, PFS, and ORR, while the majority of studies found no benefit for veliparib over standard treatment.

## 1. Introduction

Lung cancer has evolved from a rare and obscure disease to the second most common form of cancer, with the highest rate of cancer-related mortality and one of the most dismal 5-year survival rates of all cancers [1]. Lung cancer is histologically and clinically classified into small cell lung cancer (SCLC) and non–small cell lung cancer (NSCLC), which individually account for approximately 15% and 85% of lung cancer histologic subtypes, respectively, with the latter being further subcategorized into lung adenocarcinoma and squamous cell lung carcinoma [2]. Despite tremendous breakthroughs in surgical and ablative strategies, as well as chemotherapy and radiation therapy, the relative 5-year survival rates for NSCLC and SCLC remain roughly 26% and 7%, respectively, due to the scarcity of early diagnostic strategies and the poor responsiveness of currently used treatment regimens [3, 4]. This highlights the need for research into more individualized therapies. The discovery of actionable oncogenic mutations has markedly improved the treatment of many cancers, as highlighted by the progression and clinical application of targeted therapeutics hampering driver mutations [5]. Epigenetic and expression-level profiling methods have substantially enhanced our insight into the implications of the DNA-damage repair (DDR) pathway deficits and the accompanying genomic instability in tumor development and progression [6, 7].

Unlike normal cells, continuous therapeutic use of chemotherapy and/or radiation along with endogenous sources comparatively predisposes tumor cells to DNA insults, while the repairing systems are likely to be disrupted in these cells, resulting in the accumulation of mutations that drive tumor progression [8]. DDR signaling triggers the transcription and enhanced expression of repair proteins, notably poly-(ADP)-ribose polymerase (PARP), which regulate multiple DDR pathways [9]. Since these pathways are essential for the repair of DNA double-strand breaks during the S and G2 phases of the cell cycle, inhibiting the PARP enzyme tends to increase PARP immobilization at DNA single-strand breaks and the conversion of single-strand breaks to double-strand breaks, entailing homologous recombination repair for replication forks to overcome this DNA lesion [10]. According to the synthetic lethality theory, blocking both the single-strand break and the homologous recombination repair mechanisms concurrently might synergistically reduce cell viability, rendering PARP, as a fundamental component of the single-strand break, a viable therapeutic target for homologous recombination-deficient tumors [11]. Similarly, patients with homologous recombination-proficient tumors, including SCLC, can benefit from PARP inhibitors, but their effectiveness is not as striking as it is in homologous recombination-deficit tumors [12]. Furthermore, DDR mutations, such as *ATM*, *PTEN*, *MRE11*, and *FANCA* mutations, have been found in a large proportion of lung cancer patients, as well as *BRCA1/2* mutations in 5% of patients, justifying the administration of PARP inhibitors to lung cancer patients [13, 14].

Veliparib (ABT-888) is an oral selective PARP 1/2 inhibitor that has shown anticancer activity in both homologous recombination-deficit and homologous recombination-proficient tumors [15]. According to preclinical studies, veliparib sensitizes tumor cells to DNA-damaging therapies, such as chemotherapy and radiation [16]. Platinum-based chemotherapy agents, including cisplatin and carboplatin, and alkylating agents such as temozolomide, are known to have therapeutic effects in lung cancer by damaging the DNA in cancer cells and inhibiting their viability and proliferation. By combining veliparib with these chemotherapy agents, the synergistic effects may enhance the therapeutic efficacy of the chemotherapy [17]. Thus, we aimed to conduct a systematic review of the literature to evaluate the efficacy and safety of veliparib in combination with chemotherapy for the treatment of lung cancer.

## 2. Methods

This systematic review was conducted according to the Preferred Reporting Items for Systematic Reviews and Meta-Analysis 2020 guidelines [18].

### 2.1. Literature search

PubMed, Scopus, and the Web of Science databases were searched, without any time or language constraints, up to October 30, 2022. In order to find additional relevant studies, the first 30 pages of the Google Scholar search engine were manually searched [19]. Furthermore, backward and forward citation searches of all included studies were performed. The search terms used included a comprehensive combination of terms related to lung neoplasms and veliparib: (“Veliparib” OR “ABT-888” OR “NSC 737664”) AND (“Lung Neoplasms” OR “Pulmonary Blastoma” OR “Lung tumor” OR “Lung adenocarcinoma”) (S1 Table).

### 2.2. Study selection

Studies identified through the systematic search were all exported to EndNote 20 software, and any duplicates were removed. Two researchers independently screened each publication’s title and abstract using the inclusion criteria. The same two researchers then independently examined the entire texts of all studies that passed the first screening, and any disagreements were resolved via discussion or consultation with a third researcher. Only randomized controlled trials (RCTs) that evaluated the efficacy or safety of veliparib treatment in lung cancer patients, regardless of their cancer type or stage, were included in this study. Moreover, there was no minimum number of study participants for inclusion in the current study. Conversely, studies that did not meet these inclusion criteria, such as those that involved healthy individuals or patients with conditions other than lung cancer, or investigated therapeutic approaches that did not include veliparib, were excluded.

### 2.3. Data extraction

Two researchers independently performed the data extraction, using a uniform data extraction sheet in Microsoft Office Excel. The following data were extracted: 1) the study characteristics, including title, first author’s name, publication year, country of study, phase of the trial, the median length of the treatment, and the median follow-up duration; 2) the characteristics of the enrolled participants, including study population, sample size, age range, sex ratio, smoking status, Eastern Cooperative Oncology Group (ECOG) performance status, cancer ascertainment, and characteristics; and 3) the main results and safety or efficacy outcomes of the studies. All extracted data were double-checked by two other authors.

### 2.4. Quality assessment

The risk of bias and quality of the included studies were independently assessed by two researchers using version 2 of the Cochrane risk-of-bias tool for randomized trials (RoB2) [20]. The RoB2 rates each study a low, high, or unclear risk of bias (some concerns) across five domains: randomization process, deviations from the intended interventions, missing outcome data, measurement of the outcome, and selection of the reported results. Any discrepancies between the two researchers were settled via discussion or consultation with a third researcher. The risk of bias graphs were created in R software, using the robvis package [21].

## 3. Results

### 3.1. Study selection

**Fig 1:**
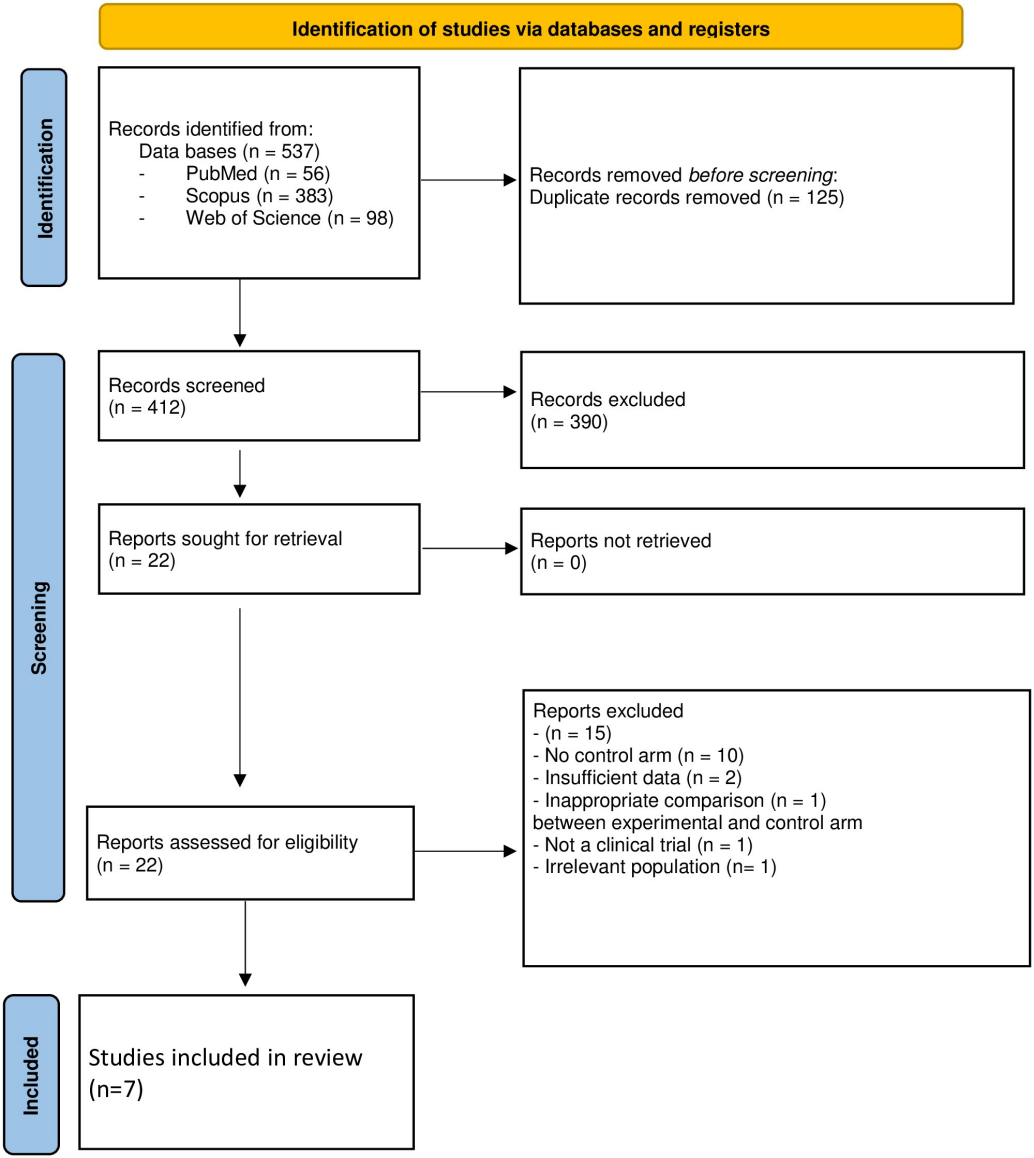
Study selection process.

In the first step, there were 537 articles identified, of which 125 were duplicates and were removed. After screening the titles and abstracts of the remaining 412 reports, another 390 reports were excluded, with the remaining 22 reports being further assessed for eligibility. Ten reports were excluded due to the absence of a control arm [22–31], two studies had insufficient data [32, 33], one study was excluded as the comparison between the intervention and control arms was inappropriate [34], one study was not a clinical trial [35], and one study was a re-analysis of a previous study [36]. After the exclusion of 15 studies, the remaining seven studies met the eligibility criteria and were included in our review [37–43] (Fig 1).

### 3.2. Study characteristics

There were 2188 patients enrolled in the seven studies, which were conducted in more than 37 countries across the globe. In one study (Argiris et al.) there were two phases, the first of which was conducted without a control arm [37]. The included studies consisted of one open-label [43], one single-blind [37], and five remaining studies were all double-blind [38–42]. The sample size of the included studies ranged from 21 to 970 participants, while the follow-up duration ranged from 10–12 weeks to four years. The median age of the participants was from 60 to 70 years and the majority of the participants were male (72.0%). In addition, most of the participants were current or former smokers (Tables 1 and 2). Each of these studies included at least one treatment arm, which involved combination therapy with veliparib, and they all detailed the simultaneous chemotherapy regimens and cycles that were employed. Carboplatin plus paclitaxel was the most commonly utilized concurrent chemotherapy treatment, followed by carboplatin plus etoposide, cisplatin plus etoposide, and the temozolomide chemotherapy regimen (Table 1). The characteristics of the included participants are summarized in Table 2.

**Table 1:** Baseline characteristics of the included studies.

Author	Year	Nation	Study type	Groups	N	Veliparib dosage	Concomitant chemotherapy	Follow-up
Argiris et al. [37]	2021 (Phase I)	USA	Single-blind RCT	VP + CT	21	Escalating doses (40, 80, 120 mg) BID for 6 weeks	Paclitaxel 45 mg/m2 followed by carboplatin once a week for 6 weeks	10–12 weeks
Argiris et al. [37]	2021 (Phase II)	USA	Single-blind RCT	VP + CT CT	18 13	120 mg BID for 6 weeks followed by 80 mg BID for 2 weeks	Paclitaxel 45 mg/m^2^ followed by carboplatin (AUC 2) once a week for 6 weeks, followed by 2 cycles of carboplatin (AUC 6), paclitaxel (200 mg/m2) every 21 days	10–12 weeks
Byers et al. [38]	2021	Multicenter (12 countries)	Double-blind RCT	VP + CT + VP VP + CT CT	61 59 61	240 mg BID for 2 weeks in a 3-week chemotherapy cycle, followed by 400 mg BID during 2 3-weeks cycle maintenance 240 mg BID for 2 weeks in a 3-week cycle chemotherapy, followed by placebo	Carboplatin (given at an AUC of 5 mg/mL/min) on Day 1 and etoposide (100 mg/ m2) on Days 1–3 of each 21-day cycle (4 cycles in total).	N/A
Govindan et al. [43]	2021	Multicenter (20 countries)	Open-label RCT	VP + CT CT	298 297	120 mg BID for 1 week in a 3-week cycle up to 6 cycles	Carboplatin (AUC 6 mg/mL/min) and paclitaxel (200 mg/m2) on Day 1 of each 21-day cycle	45.3 months 44.5 months
Owonikoko et al. [39]	2019	USA	Double-blind RCT	VP + CT CT	64 64	100 mg BID for 1 week in a 3-week cycle for 4 cycles	Cisplatin (75 mg/ m2) on day 1, etoposide (100 mg/ m2) on days 1 through 3 in a 3-week treatment cycle for 4 cycles	2 years
Pietanza et al. [40]	2018	USA	Double-blind RCT	VP + CT CT	55 49	40 mg BID for 1 week in a 4-weeks cycle	Temozolomide 200 mg/m2/day on days 1 to 5 of a 4-week cycle	N/A
Ramalingam et al. [41]	2017	Multicenter (8 countries)	Double-blind RCT	VP + CT CT	105 53	120 mg BID for 1 week of a 3-week cycle for a maximum of 6 cycle.	Carboplatin and paclitaxel on day 3 of each 3-week treatment cycle.	N/A
Ramalingam et al. [42]	2021	Multicenter (37 countries)	Double-blind RCT	VP + CT CT	486 484	120 mg BID for a week in a 3-week cycle for 6 cycles	Carboplatin (AUC 6 mg/mL/min) and paclitaxel (200 mg/ m2 on day 1 of each 3-week cycle.	4 years

Abbreviations: RCT: Randomized controlled trial; VP: Veliparib; CT: Chemotherapy; USA: United States of America; BID: Twice daily; AUC: Area under the curve; N/A: Not available.

**Table 2:** Characteristics of the participants in the studies included in the systematic review.

Author	Groups	N	Age (Median)	Sex		Histopathology					Smoking status				Performance status	
				M	F	SCC	AC	LCC	SCLC	Other	Former	Current	Never	Missing	0	1
Argiris et al. 2021 [37]	VP + CT	21	70 (53–81)	14 (67%)	7 (33%)	8 (38%)	12 (57%)	NA	NA	1 (5%)	13 (62%)	4 (19%)	4 (19%)	NA	11 (52%)	10 (48%)
Argiris et al. 2021 [37]	VP + CT CT	18 13	64 (47–78) 65 (56–75)	7 (39%) 7 (%54)	11 (61%) 6 (46%)	10 (56%) 5 (38%)	8 (44%) 8 (62%)	NA	NA	NA	9 (50%) 7 (54%)	8 (44%) 6 (46%)	1 (6%) NA	NA NA	7 (39%) 3 (23%)	11 (61%) 10 (77%)
Byers et al. 2021 [38]	VP + CT + VP VP + CT CT	61 59 61	62 (39–77) 64 (46–86) 63 (37–87)	40 (65%) 38 (64%) 38 (62%)	21 (35%) 21 (36%) 23 (38%)	NA	NA	NA	61 (100%) 59 (100%) 61 (100%)	NA	31 (51%) 32 (55%) 31 (51%)	29 (47%) 23 (40%) 27 (44%)	1 (1%) 3 (5%) 3 (5%)	NA 1 (1%) NA	21 (35%) 16 (28%) 23 (38%)	39 (65%) 42 (72%) 37 (62%)
Govindan et al. 2021 [43]	VP + CT CT	298 297	63 (27–81) 64 (34–85)	206 (69%) 207 (70%)	92 (31%) 90 (30%)	NA	Non–squamous Non–small cell lung cancer: 298 (100%) 297 (100%)		-	NA	146 (49%) 144 (48%)	152 (51%) 153 (52%)	NA	NA	116 (39%) 113 (38%)	182 (61%) 184 (62%)
Owonikoko et al. 2019 [39]	VP + CT CT	64 64	66 (59–72) 64(59–70)	34 (53%) 32 (50%)	30 (47%) 32 (50%)	NA	NA	NA	64 (100%) 64 (100%)	NA	NA	NA	NA	NA NA	15 (23%) 22 (34%)	49 (77%) 42 (66%)
Pietanza et al. 2018 [40]	VP + CT CT	55 49	63 (31–80) 62 (35–84)	24 (43%) 26 (53%)	31 (57%) 23 (47%)	NA	NA	NA	55 (100%) 49 (100%)	NA	49 (90%) 44 (90%)		3 (5%) 1 (2%)	3 (5%) 5 (8%)	16 (29%) 13 (27%)	39 (71%) 36 (73%)
Ramalingam et al.2017 [41]	VP + CT CT	105 53	63 (33–84) 62 (46–79)	75 (71%) 32 (60%)	30 (29%) 21 (40%)	51 (49%) 25 (47%)	Non–squamous Non–small cell lung cancer: 54 (51%) 28 (53%)		NA	NA	28 (27%) 14 (26%)	64 (61%) 31 (58%)	13 (12%) 8 (15%)	NA NA	35 (33%) 17 (32%)	70 (67%) 36 (68%)
Ramalingam et al. 2021 [42]	VP + CT CT	486 484	64 (36–83) 64 (33–84)	411 (85%) 384 (79%)	75 (15%) 100 (21%)	486 (100%) 484 (100%)	NA	NA	NA	NA	181 (37%) 181 (37%)	276 (57%) 276 (57%)	29 (6%) 27 (6%)	NA NA	166 (34%) 165 (34%)	320 (66%) 319 (66%)

Abbreviations: AC: Adenocarcinoma; AUC: Area under the curve; BID: Twice daily; CT: Chemotherapy; F: Female; LCC: Large-cell carcinoma; M: Male; N: Number; NA: Not available; SCC: Squamous cell carcinoma; SCLC: Small cell lung cancer; USA: United States of America; VP: Veliparib.

### 3.3. Assessment of risk of bias

**Fig 2:**
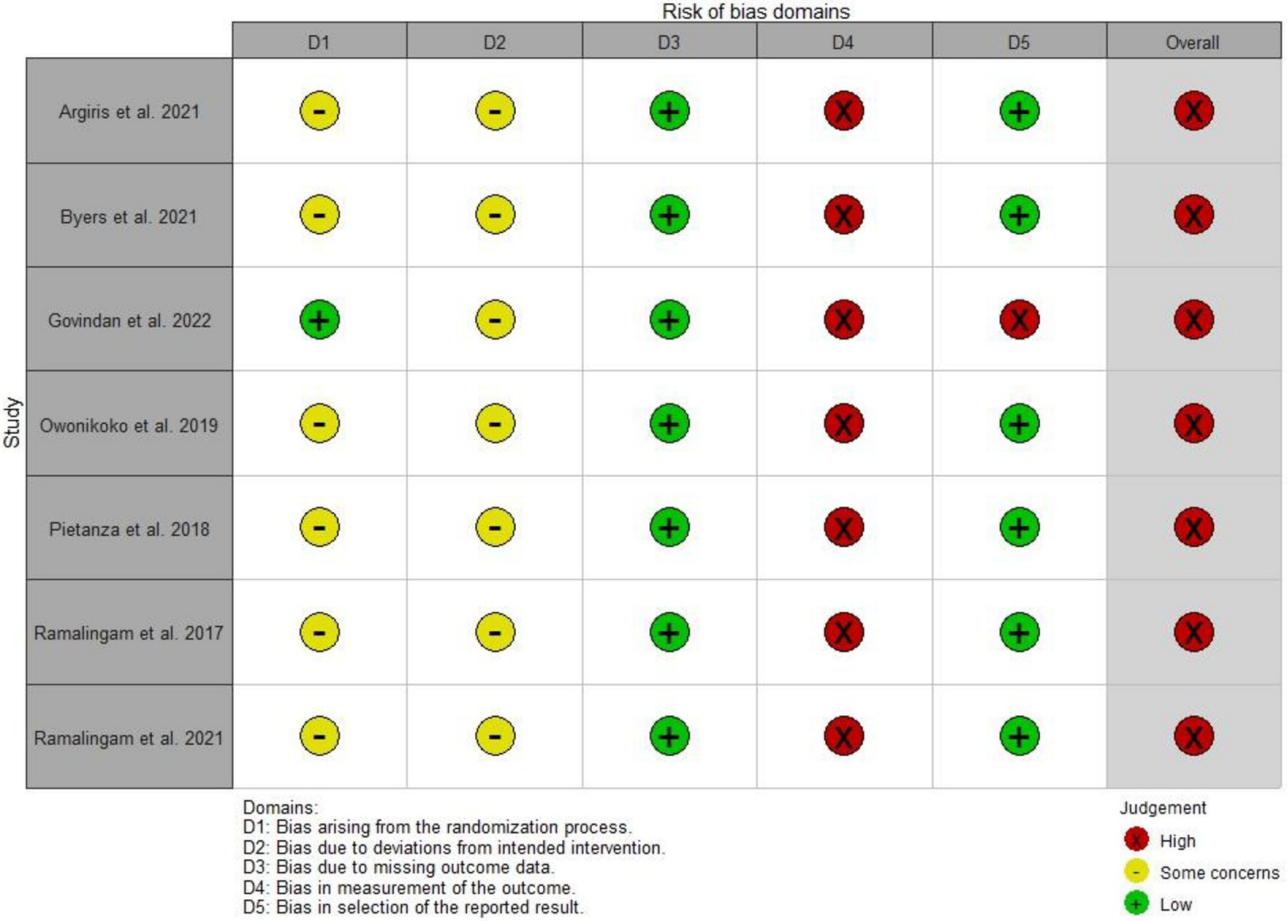
Summary of risk of bias assessment for the included studies.

All of the included studies were found to have a high overall risk of bias, with a high risk of bias being noted in the measurement of outcomes in all studies. However, all studies had a low risk of bias in the missing outcome data. In addition, the majority of the included studies [37–42] (all but one) [43] were rated as having a low risk of bias in the selection of the reported results (Fig 2 and S2 Table).

### 3.4. Efficacy

Survival outcomes were the primary endpoints in the included studies. All of these studies reported progression-free survival (PFS), which only improved in two of the studies [38, 39]. The PFS was similar between the chemotherapy plus veliparib and the chemotherapy alone arms in the five remaining studies. The study conducted by Byers et al. included three arms: veliparib plus chemotherapy followed by veliparib maintenance (i.e., veliparib throughout), veliparib plus chemotherapy followed by a placebo (i.e., veliparib combination-only), or a placebo plus chemotherapy followed by a placebo (i.e., control arm). As the authors of the study concluded, there was a statistically significant difference in PFS only between the veliparib throughout and control arm (p = 0.06; level of significant: p<0.2), but PFS did not differ between the veliparib combination-only and the control arm (p = 0.92) [38].

The overall survival (OS) was reported in all included studies, but only Ramalingam et al. 2021 [42] found a statistically significant difference between the treatment and control arms. Furthermore, objective response rate (ORR) and the duration of response (DoR) were the other outcomes that were measured in all and five [38, 40–43] of the included studies, respectively. The ORR only favored the intervention arm in one study [40]. Moreover, adding veliparib to a conventional chemotherapy regimen did not increase the DoR in any of the studies (Table 3).

**Table 3:** Efficacy of the treatments in the included studies.

Study ID		Efficacy							
		Intervention				Control			
		ORR	PFS	OS	DoR	ORR	PFS	OS	DoR
Argiris et al. (2021) [37]	Phase 1	**Outcomes from initial registration (Start of CRT)**: N = 18, 86% (64%-97%)	**Outcomes from initial registration (Start of CRT)**: Median in months (95% CI): 11.5 (9.5, 19.2) 1-year estimate (95% CI): 40% (19%, 60%) **Outcomes from start of consolidation**: 1-year estimate (95% CI): 50% (23%, 72%	**Outcomes from initial registration (Start of CRT)**: Median in months (95% CI): 32.9 (13.8, 37.8) 1-year estimate: 70% (45%, 85%) **Outcomes from start of consolidation**: 1-year estimate: 79% (47%, 93%)	NA	NA	NA	NA	NA
	Phase 2	**Outcomes from initial registration (Start of CRT)**: N = 10, 56% (31%, 78%)	**Outcomes from initial registration (Start of CRT)**: HR (95% CI): 1.47 (0.59, 3.66) Median in months (95% CI): 9.3 (7.3, 17.4) 1-year estimate (95% CI): 41% (18%, 63%) **Outcomes from start of consolidation**: HR (95% CI): 1.65 (0.54, 5.01) 1-year estimate (95% CI): 43% (16%, 68%)	**Outcomes from initial registration (Start of CRT)**: HR (95% CI): 0.65 (0.24, 1.75) Median in months (95% CI): 27.6 (17.4, 27.6) 1-year estimate: 89% (61%, 97%) **Outcomes from start of consolidation**: HR (95% CI): 0.71 (0.23, 2.20) 1-year estimate: 76% (42%, 91%)	NA	**Outcomes from initial registration (Start of CRT)**: N = 9, 69% (38%, 91%)	**Outcomes from initial registration (Start of CRT)**: HR (95% CI): 0.20 Median in months (95% CI): 9.9 (5.7, 23.6) 1-year estimate (95% CI): 46% (19%, 70%) **Outcomes from start of consolidation**: HR (95% CI): 0.19 1-year estimate (95% CI): 40% (12%, 67%)	**Outcomes from initial registration (Start of CRT)**: HR (95% CI): 0.19 Median in months (95% CI): 15.2 (6.6, 20.6) 1-year estimate: 54% (25%, 76%) **Outcomes from start of consolidation**: HR (95% CI): 0.28 1-year estimate: 50% (18%, 76%)	NA
Byers et al. (2021) [38]		**Throughout**: 77% (CR: 3.3%) **Combination**: 59% (CR: 3.4%)	Median in months **Throughout**: 5.8 **Combination**: 5.7	Median in months **Throughout**: 10.1 **Combination**: 10.0	Median in months **Throughout**: 4.7 **Combination**: 4.3	64% (CR: 3.3%) no statistically significant differences in ORR were observed between treatment arms	Median in months: 5.6	Median in months: 12.4	Median in months: 5.3
Govindan et al. (2021) [43]		78 (26) CR: 2 (1)	5.9 months (5.0–6.5) (HR: 1.035 [95% CI: 0.867–1.235]; nominal 2- sided P = 0.473)	11.2 months (HR: 0.644 [95% CI: 0.396–1.048], stratified log-rank 2-sided P = .113)	7.3 months	86 (29) CR: 2 (1)	6.7 months (5.6–7.2)	9.2 months	6.6 months
Owonikoko et al. (2019) [39]		71.9%; one-sided Fisher P = 0.29	6.1 months (95% CI, 5.9 to 6.7 months)	10.3 months (95% CI, 8.9–12.0 months)	NA	65.6%; one-sided Fisher P = 0.29	8.9 months (95% CI, 8.3–11.3 months)	8.9 months (95% CI, 8.3–11.3 months)	NA
Pietanza et al. (2018) [40]		39%; 95% CI, 25%-54% P = 0.016	3.8 months (log-rank P = .39; hazard ratio, 0.84; 95% CI, 0.56–1.25)	8.2 months (95% CI, 6.4–12.2 months; P = 0.50)	4.61 months (95% CI, 2.86–9.9 months) (N = 19)	14%; 95% CI, 5% to 27%; P = 0.016	2.0 months	7.0 months (95% CI, 5.3 to 9.5 months; P = 0.50)	3.68 months (95% CI, 2.76 months to not achieved) (N = 6)
Ramalingam et al. (2017) [41]		32.4%; 95% CI, 23.6–42.2	5.8 months (95% CI, 4.3–6.5) (HR, 0.72; 95% CI, 0.45–1.15) **In squamous cell histology**: 6.5 months (95% CI, 4.4–8.4) (HR, 0.54; 95% CI, 0.26–1.12) **In non-squamous histology**: There was no difference in median PFS between the 2 groups (HR, 0.87; 95% CI, 0.48–1.59)	11.7 months; 95% CI, 8.8–13.7 HR, 0.80 (95% CI, 0.54–1.18; P 1⁄4 0.27) **In squamous cell histology**: 10.3 months; 95% CI, 8.3–13.2 (HR, 0.73; 95% CI, 0.43–1.24) **In non-squamous histology**: 12.8 months; 95% Cl, 8.0–17.2 HR, 0.90; 95% cl, 0.51–1.58	6.9 months (95% CI, 4.5–7.0) (HR, 0.47; 95% CI, 0.16–1.42)	32.1%; 95% CI, 19.9–46.3	4.2 months (95% CI, 3.1–5.6) **In squamous cell histology**: 4.1 months (95% CI, 2.8–NA) **In non-squamous histology**: There was no difference in median PFS between the 2 groups (HR, 0.87; 95% CI, 0.48–1.59)	9.1 months; 95% CI, 5.4–12.3 **In squamous cell histology**: 8.4 months; 95% CI, 5.0–12.9 11.1 months; 95% Cl, 4.8–14.6	4.3 months (95% CI, 2.8–not available)
Ramalingam et al. (2021) [42]		0.37	**In the ITT population**: 5.6 months; 95% Cl, 5.6–5.8 HR, 0.897; 95% Cl, 0.779–1.032; stratified log-rank P, 0.107	11.9 months; 95% Cl, 10.5–13.5 HR, 0.905; 95% Cl, 0.744–1.101; stratified log-rank P, 0.266 **In the ITT population**: 12.2 months; 95% Cl, 10.9–13.5 HR, 0.853; 95% Cl, 0.747–0.976; stratified log-rank P, 0.032	**Among patients who achieved an overall response (N = 180 per arm)**: 5.4 months	0.37	**In the ITT population**: 12.2 months; 95% Cl, 5.5–5.7	11.1 months; 95% Cl, 9.6 to 12.6 **In the ITT population**: 11.2 months; 95% Cl, 10.1–12.6	Among patients who achieved an overall response (N = 180 per arm): 5.5 months

Abbreviations: CI: Confidence interval; CRT: Chemoradiotherapy; DoR: Duration of response; HR: Hazard ratio; ITT: Intent-to-treat; NA: Not available; N: Number; ORR: Objective response rate; OS: Overall survival; PFS: Progression-free survival; CR: Complete response.

In two studies [42, 43], tissue samples were taken to evaluate the level of the LP52 biomarker, and a subsequent subgroup analysis was done based on the presence or absence of this biomarker. Although in the studies by Govindan et al. [43] and Ramalingam et al. 2021 [42] the participants in the two arms had statistically similar PFS and OS, respectively, those who had positive LP52 biomarkers showed improved efficacy.

### 3.5. Safety

All studies defined adverse events (AEs) using the National Cancer Institute-Common Terminology Criteria for Adverse Events (NCI-CTCAE), version 4.0. Moreover, treatment-related AEs were reported in both the intervention and control arms across all studies. Hematologic AEs, including anemia, neutropenia, lymphopenia, leukopenia, thrombocytopenia, and febrile neutropenia were the most commonly reported treatment-related AEs, followed by non-hematologic AEs such as fatigue, nausea, vomiting, dizziness, dermatologic AEs (e.g., alopecia and dry skin), myalgia, arthralgia, constipation, diarrhea, and dyspnea. The most common AEs reported in the control arms were fatigue, nausea, constipation, anemia, neutropenia, and thrombocytopenia. Most of the deaths were not related to the received treatments. Four cases of grade-5 treatment-related AEs were reported in two of the studies [37, 38] (Table 4).

**Table 4:** Reported adverse events among the included studies.

Study ID		Adverse event (%)¶		
		Intervention		Control
Argiris et al. (2021) [37]	Phase 1	Chemoradiation therapy	Grade 4 lymphopenia (14%) Grade 4 neutropenia (5%) Grade 3 AEs (57%), which were mostly hematologic. Treatment-related death (5%) due to esophageal perforation 8 months after completing chemoradiation therapy	NA
		Consolidation therapy	Grade 4 neutropenia (21%) Treatment related death (7%) due to neutropenic sepsis	NA
	Phase 2	Chemoradiation therapy	Grade 4 lymphopenia (12%) Grade 3 AEs (35%)	Grade 4 hypoglycemia (6%) Grade 4 lymphopenia (12%) Grade 3 AEs (46%)
		Consolidation therapy	Grade 4 neutropenia (16%) Grade 4 lymphopenia (8%) Grade 3 AEs (42%)	Grade 5 pneumonitis followed by grade 4 lymphopenia, which led to death (10%) Grade 4 hyperglycemia (10%) Grade 3 AEs (10%)
Byers et al. (2021) [38]		Veliparib throughout	Grade 3/4 AEs (82%) Serious AEs (55%) Febrile neutropenia (8%) Thrombocytopenia (5%) Pneumonia (10%) Treatment-related death (11%)	Grade 3/4 AEs (68%) Serious AEs (45%) Febrile neutropenia (5%) Thrombocytopenia (3%) Pneumonia (0%) Treatment-related death (8%)
		Veliparib combination-only	Grade 3/4 AEs (88%) Serious AEs (67%) Febrile neutropenia (12%) Thrombocytopenia (10%) Pneumonia (2%) Treatment-related death (17%)	NA
Govindan et al. (2021) [43]		Grade 3/4 AEs (68%) Serious AEs (41%) Grade 3/4 alopecia (<1%) Grade 3/4 peripheral sensory neuropathy (5%) Grade 3/4 anemia (17%) Grade 3/4 neutropenia (29%) Grade 3/4 nausea (2%) Grade 3/4 fatigue (2%) Grade 3/4 thrombocytopenia (7%) Grade 3/4 constipation (1%) Grade 3/4 decreased appetite (2%) Grade 3/4 diarrhea (2%) Grade 3/4 dyspnea (3%) Grade 3/4 leukopenia (7%) Grade 3/4 vomiting (1%) Grade 3/4 arthralgia (2%) Grade 3/4 myalgia (1%) Grade 3/4 insomnia (0%) Grade 3/4 asthenia (1%) Grade 3/4 cough (<1%) Grade 3/4 pneumonia (6%) Grade 3/4 stomatitis (<1%) Grade 3/4 febrile neutropenia (5%) Grade 3/4 pulmonary embolism (2%)		Grade 3/4 AEs (57%) Serious AEs (34%) Grade 3/4 alopecia (0%) Grade 3/4 peripheral sensory neuropathy (1%) Grade 3/4 anemia (16%) Grade 3/4 neutropenia (18%) Grade 3/4 nausea (2%) Grade 3/4 fatigue (3%) Grade 3/4 thrombocytopenia (9%) Grade 3/4 constipation (0%) Grade 3/4 decreased appetite (3%) Grade 3/4 diarrhea (1%) Grade 3/4 dyspnea (3%) Grade 3/4 leukopenia (5%) Grade 3/4 vomiting (2%) Grade 3/4 arthralgia (1%) Grade 3/4 myalgia (<1%) Grade 3/4 insomnia (<1%) Grade 3/4 asthenia (2%) Grade 3/4 cough (<0%) Grade 3/4 pneumonia (7%) Grade 3/4 stomatitis (1%) Grade 3/4 febrile neutropenia (2%) Grade 3/4 pulmonary embolism (5%)
Owonikoko et al. (2019) [39]		Anemia: grade 3 (17%), grade 4 (2%) Febrile neutropenia: grade 3 (5%) Fatigue: grade 3 (3%) Lymphopenia: grade 3 (8%) Neutropenia: grade 3 (20%), grade 4 (29%) Leukopenia: grade 3 (8%), grade 4 (11%) Dehydration: grade 3 (5%), grade 4 (2%) Hyperglycemia: grade 3 (5%) Hyponatremia: grade 3 (12%) Acute kidney injury: grade 3 (5%)		Anemia: grade 3 (12%) Febrile neutropenia: grade 3 (5%), grade 5 (2%) Fatigue: grade 3 (5%) Lymphopenia: (0%) Neutropenia: grade 3 (14%), grade 4 (18%) Leukopenia: grade 3 (12%), grade 4 (2%) Dehydration: grade 3 (3%) Hyperglycemia: (0%) Hyponatremia: grade 3 (2%), grade 4 (5%) Acute kidney injury: grade 3 (2%), grade 4 (2%)
Pietanza et al. (2018) [40]		Anemia: Grade 1/2 (43%), Grade 3/4 (11%) Leukopenia: Grade 1/2 (30%), Grade 3/4 (24%) Lymphopenia: Grade 1/2 (15%), Grade 3/4 (20%) Neutropenia: Grade 1/2 (11%), Grade 3/4 (31%) Febrile Neutropenia: Grade 1/2 (0%), Grade 3/4 (4%) Thrombocytopenia: Grade 1/2 (24%), Grade 3/4 (50%) Alkaline phosphatase increase: Grade 1/2 (15%), Grade 3/4 (0%) Anorexia: Grade 1/2 (19%), Grade 3/4 (0%) Constipation: Grade 1/2 (17%), Grade 3/4 (2%) Dermatologic: Grade 1/2 (11%), Grade 3/4 (0%) Dizziness: Grade 1/2 (11%), Grade 3/4 (0%) Fatigue: Grade 1/2 (44%), Grade 3/4 (4%) Nausea: Grade 1/2 (41%), Grade 3/4 (0%) Vomiting: Grade 1/2 (17%), Grade 3/4 (0%)		Anemia: Grade 1/2 (41%), Grade 3/4 (2%) Leukopenia: Grade 1/2 (17%), Grade 3/4 (7%) Lymphopenia: Grade 1/2 (11%), Grade 3/4 (26%) Neutropenia: Grade 1/2 (0%), Grade 3/4 (7%) Febrile Neutropenia: Grade 1/2 (0%), Grade 3/4 (0%) Thrombocytopenia: Grade 1/2 (33%), Grade 3/4 (9%) Alkaline phosphatase increase: Grade 1/2 (4%), Grade 3/4 (0%) Anorexia: Grade 1/2 (11%), Grade 3/4 (0%) Constipation: Grade 1/2 (24%), Grade 3/4 (0%) Dermatologic: Grade 1/2 (7%), Grade 3/4 (0%) Dizziness: Grade 1/2 (2%), Grade 3/4 (0%) Fatigue: Grade 1/2 (43%), Grade 3/4 (4%) Nausea: Grade 1/2 (35%), Grade 3/4 (0%) Vomiting: Grade 1/2 (13%), Grade 3/4 (2%)
Ramalingam et al. (2017) [41]		Serious AEs (27%) Grade ≥3 Neutropenia (19%) Grade ≥3 Anemia (10%) Grade ≥3 Alopecia (7%) Grade ≥3 Leukopenia (6%) Grade ≥3 Thrombocytopenia (5%) Grade ≥3 Nausea (4%) Grade ≥3 Hyperkalemia (4%) Grade ≥3 Arthralgia (3%) Grade ≥3 Fatigue (3%) Grade ≥3 Hypersensitivity (3%) Grade ≥3 Hyponatremia (2%) Grade ≥3 Myalgia (2%) Grade ≥3 Weight loss (2%)		Serious AEs (23%) Grade ≥3 Neutropenia (23%) Grade ≥3 Anemia (10%) Grade ≥3 Alopecia (6%) Grade ≥3 Leukopenia (0%) Grade ≥3 Thrombocytopenia (6%) Grade ≥3 Nausea (0%) Grade ≥3 Hyperkalemia (2%) Grade ≥3 Arthralgia (0%) Grade ≥3 Fatigue (0%) Grade ≥3 Hypersensitivity (0%) Grade ≥3 Hyponatremia (2%) Grade ≥3 Myalgia (0%) Grade ≥3 Weight loss (0%)
Ramalingam et al. (2021) [42]		Grade ≥ 3 AEs (60%) Serious AEs (32%) Grade 3/4 anemia (10%) Grade 3/4 neutropenia (24%) Grade 3/4 thrombocytopenia (6%)		Grade ≥ 3 AEs (58%) Serious AEs (34%) Grade 3/4 anemia (11%)

^¶^Adverse events were defined using the National Cancer Institute-Common Terminology Criteria for Adverse Events (NCI-CTCAE), version 4.0.

Abbreviations: AE: Adverse event; NA: not available.

## 4. Discussion

The present qualitative synthesis of the seven RCTs showed that in most studies there were no statistically significant differences between those who received veliparib and the controls, in terms of OS, PFS, ORR, and DoR. Regarding the safety profile, the frequency of any grade and severe grade AEs were generally higher in the intervention group containing veliparib, than among the controls.

The use of PARP inhibitors alone, or in combination with other regimes, can be used for SCLC management for repairing DNA damage, inhibiting DNA damage, or activating the immune system [44]. Most of the previous studies using PARP inhibitors to treat SCLC used olaparib or veliparib and demonstrated modest efficacy [45]. The efficacy measures that were most frequently reported in the included studies were OR, PFS, and ORR. The meta-analysis by Bao and colleagues on PARP inhibitors in cancer therapy showed that PARP inhibitors significantly increased the PFS (HR: 0.67; 95% CI: 0.50–0.90) [46]. However, PARP inhibitors had no significant effect on PFS (HR: 0.98; 95% CI: 0.83–1.15) or OS (HR: 1.00; 95% CI: 0.76–1.31) among lung cancer patients [46]. Another recent meta-analysis on the efficacy of PARP inhibitors for treating solid tumors found that PARP inhibitors did not improve the OS and ORR for NSCLC and SCLC (p<0.05), while it only improved PFS in SCLC (HR: 0.77; 95% CI: 0.63–0.95) [47]. The subgroup analysis by type of PARP inhibitor showed that veliparib can significantly improve the PFS (HR: 0.82; 95% CI: 0.80–0.97), while it did not reveal any statistically significant improvement in ORR (HR: 1.04; 95% CI: 0.89–1.22) or OR (HR: 0.93; 95% CI: 0.83–1.05) [47]. Similarly, we found that only two studies reported improvements in PFS and only one study reported improvements in OS or ORR. One of the limitations of meta-analyses is that the analyses are likely to lead to insignificant results when there are a small number of primary studies available. Therefore, the meta-analyses should be re-run in the future with a larger number of studies.

The efficacy and safety of PARP inhibitors have also been investigated for other types of cancer. The results of a network meta-analysis showed significantly improved PFS (HR: 0.37; 95% CI: 0.20–0.69) and ORR (HR: 7.07; 95% CI: 1.83–27.32) for veliparib + chemotherapy, compared with chemotherapy alone, while it was not significant in terms of pathologic complete response (HR: 2.06; 95% CI: 0.84–5.07) [48]. Moreover, PARP inhibitors have also been used to treat prostate cancer, although veliparib has been found to be the least potent of the PARPs evaluated [49]. For ovarian cancer, PARP inhibitors significantly improved PFS (HR: 0.51; 95% CI: 0.40–0.65), when compared with a placebo or chemotherapy alone [50]. The differences between the findings on the efficacy of PARP inhibitors, in particular veliparib, for different solid tumors may be as a result of variations in the inclusion/exclusion criteria, types of analyses used, and the number of studies included.

The descriptive results on the frequency of AEs in patients with lung cancer revealed an overall higher frequency of any grade and severe grade AEs in those receiving veliparib plus chemotherapy, compared with those receiving standard chemotherapy. Interestingly, a study by Bao et al. on the safety of PARP inhibitors in treating cancers, reported a decreased risk of asthenia (RR: 0.34; 95% CI: 0.14–0.82) and an increased risk of neutropenia (RR: 1.14; 95% CI: 1.01–1.29), while there were no differences between the intervention and control groups for other respiratory, gastrointestinal, and hematologic AEs [46]. A systematic review of trials among patients with advanced ovarian cancer showed that those receiving PARP inhibitors had a significantly higher risk of hematologic and gastrointestinal AEs [51]. Overall, it seems that patients with different types of cancer, in particular lung cancer, who receive PARP inhibitors might have a higher frequency of any grade and severe grade AEs, compared with those on standard chemotherapy, although this should be further investigated in future RCTs and meta-analyses.

The studies included in our systematic review received low-quality ratings, using the last version of the Cochrane RoB2 tool. In contrast, a systematic review and meta-analysis on PARP inhibitors in solid tumors, which included 29 studies, showed a low risk of bias in most domains and the only domain with a high risk of bias was performance bias, due to the inclusion of open-label studies [47]. These differences can be explained by the use of different quality rating tools (version 1 vs. version 2 of the Cochrane risk of bias assessment) and the evaluation of different studies [47]. A study by Chang et al., which evaluated the efficacy and safety of PARP inhibitors for treating breast cancer, also used the Cochrane RoB2 for quality assessment [52]. They found that two of the six trials had a high risk of bias, which was due to the missing outcome data domain [52]. In addition, in a meta-analysis of studies using PARP inhibitors as maintenance therapy for ovarian cancer, a low risk of bias was found in all six of the included RCTs using the Cochrane RoB2 [53]. The high risk of bias among the included studies in our systematic review was mostly due to the measurement of the outcome domain. Therefore, it is of great importance to conduct further high-quality RCTs for treating lung cancer patients with veliparib, with specific attention to the deviation from intended interventions and the measurement of outcomes. The high risk of bias in the studies included in our systematic review should be noted in the interpretation and generalization of the study outcomes.

The safety and efficacy of PARP inhibitors have been previously evaluated in patients with several different types of cancer [46, 47]. However, to the best of our knowledge, this is the first study that has specifically focused on veliparib in patients with lung cancer. However, the current systematic review has several limitations that should be taken into consideration when interpreting the results. Firstly, the number of included studies is relatively small, so the findings should be interpreted with some caution. Secondly, due to the heterogeneity between the studies, especially in terms of the interventions and subjects in the control group, a meta-analysis and sub-group analysis could not be performed. Thirdly, we searched three online databases, in addition to grey literature, but there is still the possibility that some eligible studies were missed. Fourthly, all of the included studies had a high risk of bias, which also highlights the need to interpret the data with some caution. Fifthly, due to the limited number of studies, we could not evaluate selection or publication bias. In addition, although we mentioned the demographic and clinical characteristics of participants, there might be other confounding variables that were not evaluated. Finally, this research can be seen as a guide to further robust research on the clinical use of veliparib as a PARP inhibitor in patients with lung cancer.

## 5. Conclusion

Although veliparib has been shown to improve the OS, PFS, and ORR in a small number of studies, for the majority there were no significant differences between the intervention and control arms. In addition, veliparib plus chemotherapy showed a higher rate of AEs than did standard chemotherapy for lung cancer. There is a critical need for additional high-quality clinical trials on the safety and efficacy of veliparib in lung cancer patients. Upon completion of these studies, a meta-analysis would also be recommended.

## Supporting information

S1 Table Search strategy for PubMed, Scopus, Web of Science and Google Scholar. (DOCX)

S2 Table Quality assessment of the included studies. (DOCX)

S1 Checklist PRISMA 2020 checklist. (DOCX)

## Abbreviations

AE: adverse event
CI: confidence interval
DDR: DNA damage repair
DoR: Duration of response
ECOG: Eastern Cooperative Oncology Group
HR: hazard ratio
NSCLC: non-small cell lung cancer
ORR: overall response rate
OS: overall survival
PARP: poly-ADP ribose polymerase
PFS: progression-free survival
RCT: randomized controlled trial
RoB2: risk of bias 2
SCLC: small cell lung cancer

## References

[1] Dela Cruz CS, Tanoue LT, Matthay RA. Lung cancer: epidemiology, etiology, and prevention. Clin Chest Med. 2011;32(4):605–44. doi: 10.1016/j.ccm.2011.09.001 . 22054876 PMC3864624

[2] Shahverdi M, Hajiasgharzadeh K, Sorkhabi AD, Jafarlou M, Shojaee M, Jalili Tabrizi N, et al. The regulatory role of autophagy-related miRNAs in lung cancer drug resistance. Biomedicine & Pharmacotherapy. 2022;148:112735. doi: 10.1016/j.biopha.2022.112735 35193040

[3] Oze I, Hotta K, Kiura K, Ochi N, Takigawa N, Fujiwara Y, et al. Twenty-seven years of phase III trials for patients with extensive disease small-cell lung cancer: disappointing results. PLoS One. 2009;4(11):e7835. Epub 20091113. doi: 10.1371/journal.pone.0007835 . 19915681 PMC2773043

[4] Grzywacz VP, Quinn TJ, Almahariq MF, Siddiqui ZA, Kim SW, Guerrero TM, et al. Trimodality therapy for patients with stage III non-small-cell lung cancer: A comprehensive surveillance, epidemiology, and end results analysis. Cancer Treat Res Commun. 2022;32:100571. Epub 20220502. doi: 10.1016/j.ctarc.2022.100571 . 35533588

[5] Lim ZF, Ma PC. Emerging insights of tumor heterogeneity and drug resistance mechanisms in lung cancer targeted therapy. J Hematol Oncol. 2019;12(1):134. Epub 20191209. doi: 10.1186/s13045-019-0818-2 . 31815659 PMC6902404

[6] O’Connor MJ. Targeting the DNA Damage Response in Cancer. Mol Cell. 2015;60(4):547–60. doi: 10.1016/j.molcel.2015.10.040 . 26590714

[7] Groelly FJ, Fawkes M, Dagg RA, Blackford AN, Tarsounas M. Targeting DNA damage response pathways in cancer. Nature Reviews Cancer. 2022. doi: 10.1038/s41568-022-00535-5 36471053

[8] Zhou J, Zhou XA, Zhang N, Wang J. Evolving insights: how DNA repair pathways impact cancer evolution. Cancer Biol Med. 2020;17(4):805–27. Epub 20201215. doi: 10.20892/j.issn.2095-3941.2020.0177 . 33299637 PMC7721097

[9] Roos WP, Thomas AD, Kaina B. DNA damage and the balance between survival and death in cancer biology. Nat Rev Cancer. 2016;16(1):20–33. Epub 20151218. doi: 10.1038/nrc.2015.2 . 26678314

[10] Noël G, Giocanti N, Fernet M, Mégnin-Chanet F, Favaudon V. Poly(ADP-ribose) polymerase (PARP-1) is not involved in DNA double-strand break recovery. BMC Cell Biol. 2003;4:7. Epub 20030716. doi: 10.1186/1471-2121-4-7 . 12866953 PMC179890

[11] Xu X, Nowsheen S, Deng M. Exploring the DNA damage response pathway for synthetic lethality. Genome Instability & Disease. 2022. doi: 10.1007/s42764-022-00087-w

[12] Pilié PG, Gay CM, Byers LA, O’Connor MJ, Yap TA. PARP Inhibitors: Extending Benefit Beyond BRCA-Mutant Cancers. Clin Cancer Res. 2019;25(13):3759–71. Epub 20190213. doi: 10.1158/1078-0432.CCR-18-0968 . 30760478

[13] Judd J, Abdel Karim N, Khan H, Naqash AR, Baca Y, Xiu J, et al. Characterization of KRAS Mutation Subtypes in Non-small Cell Lung Cancer. Mol Cancer Ther. 2021;20(12):2577–84. Epub 20210913. doi: 10.1158/1535-7163.MCT-21-0201 . 34518295 PMC9662933

[14] Rose M, Burgess JT, O’Byrne K, Richard DJ, Bolderson E. PARP Inhibitors: Clinical Relevance, Mechanisms of Action and Tumor Resistance. Front Cell Dev Biol. 2020;8:564601. Epub 20200909. doi: 10.3389/fcell.2020.564601 . 33015058 PMC7509090

[15] Pommier Y, O’Connor MJ, de Bono J. Laying a trap to kill cancer cells: PARP inhibitors and their mechanisms of action. Sci Transl Med. 2016;8(362):362ps17. doi: 10.1126/scitranslmed.aaf9246 . 27797957

[16] Donawho CK, Luo Y, Luo Y, Penning TD, Bauch JL, Bouska JJ, et al. ABT-888, an orally active poly(ADP-ribose) polymerase inhibitor that potentiates DNA-damaging agents in preclinical tumor models. Clin Cancer Res. 2007;13(9):2728–37. doi: 10.1158/1078-0432.CCR-06-3039 . 17473206

[17] Burgess JT, Rose M, Boucher D, Plowman J, Molloy C, Fisher M, et al. The Therapeutic Potential of DNA Damage Repair Pathways and Genomic Stability in Lung Cancer. Frontiers in oncology. 2020;10:1256. Epub 2020/08/28. doi: 10.3389/fonc.2020.01256 . 32850380 PMC7399071

[18] Page MJ, McKenzie JE, Bossuyt PM, Boutron I, Hoffmann TC, Mulrow CD, et al. The PRISMA 2020 statement: an updated guideline for reporting systematic reviews. Bmj. 2021;372:n71. Epub 2021/03/31. doi: 10.1136/bmj.n71 . 33782057 PMC8005924

[19] Haddaway NR, Collins AM, Coughlin D, Kirk S. The Role of Google Scholar in Evidence Reviews and Its Applicability to Grey Literature Searching. PLOS ONE. 2015;10(9):e0138237. doi: 10.1371/journal.pone.0138237 26379270 PMC4574933

[20] Sterne JAC, Savović J, Page MJ, Elbers RG, Blencowe NS, Boutron I, et al. RoB 2: a revised tool for assessing risk of bias in randomised trials. Bmj. 2019;366:l4898. Epub 2019/08/30. doi: 10.1136/bmj.l4898 . 31462531

[21] McGuinness LA, Higgins JPT. Risk-of-bias VISualization (robvis): An R package and Shiny web app for visualizing risk-of-bias assessments. Res Synth Methods. 2021;12(1):55–61. Epub 2020/04/27. doi: 10.1002/jrsm.1411 . 32336025

[22] Kummar S, Kinders R, Gutierrez ME, Rubinstein L, Parchment RE, Phillips LR, et al. Phase 0 clinical trial of the poly (ADP-ribose) polymerase inhibitor ABT-888 in patients with advanced malignancies. Journal of Clinical Oncology. 2009;27(16):2705–11. doi: 10.1200/JCO.2008.19.7681 19364967 PMC2739635

[23] Owonikoko TK, Dahlberg SE, Khan SA, Gerber DE, Dowell J, Moss RA, et al. A phase 1 safety study of veliparib combined with cisplatin and etoposide in extensive stage small cell lung cancer: A trial of the ECOG-ACRIN Cancer Research Group (E2511). Lung Cancer. 2015;89(1):66–70. doi: 10.1016/j.lungcan.2015.04.015 25985977 PMC4539011

[24] Mizugaki H, Yamamoto N, Nokihara H, Fujiwara Y, Horinouchi H, a S, et al. A phase 1 study evaluating the pharmacokinetics and preliminary efficacy of veliparib (ABT-888) in combination with carboplatin/paclitaxel in Japanese subjects with non-small cell lung cancer (NSCLC). Cancer Chemother Pharmacol. 2015;76(5):1063–72. doi: 10.1007/s00280-015-2876-7 26433581 PMC4612330

[25] Atrafi F, Groen HJM, Byers LA, Garralda E, Lolkema MP, Sangha RS, et al. A Phase I Dose-Escalation Study of Veliparib Combined with Carboplatin and Etoposide in Patients with Extensive-Stage Small Cell Lung Cancer and Other Solid Tumors. Clinical Cancer Research. 2019;25(2):496–505. doi: 10.1158/1078-0432.CCR-18-2014 30327308

[26] LoRusso PM, Li J, Burger A, Heilbrun LK, Sausville EA, Boerner SA, et al. Phase I safety, pharmacokinetic, and pharmacodynamic study of the poly(ADP-ribose) polymerase (PARP) inhibitor veliparib (ABT-888) in combination with irinotecan in patients with advanced solid tumors. Clinical Cancer Research. 2016;22(13):3227–37. doi: 10.1158/1078-0432.CCR-15-0652 26842236 PMC4930710

[27] Stoller R, Schmitz JC, Ding F, Puhalla S, Belani CP, Appleman L, et al. Phase I study of veliparib in combination with gemcitabine. Cancer Chemotherapy and Pharmacology. 2017;80(3):631–43. doi: 10.1007/s00280-017-3409-3 28770300 PMC5734661

[28] Villalona-Calero MA, Duan WR, Zhao WQ, Shilo K, Schaaf LJ, Thurmond J, et al. Veliparib Alone or in Combination with Mitomycin C in Patients with Solid Tumors With Functional Deficiency in Homologous Recombination Repair. Jnci-Journal of the National Cancer Institute. 2016;108(7):10. doi: 10.1093/jnci/djv437 26848151 PMC4948564

[29] Clarke JM, Patel JD, Robert F, Kio EA, Thara E, Camidge DR, et al. Veliparib and nivolumab in combination with platinum doublet chemotherapy in patients with metastatic or advanced non-small cell lung cancer: A phase 1 dose escalation study. Lung Cancer. 2021;161:180–8. doi: 10.1016/j.lungcan.2021.09.004 34607210

[30] Kozono DE, Stinchcombe TE, Salama JK, Bogart J, Petty WJ, Guarino MJ, et al. Veliparib in combination with carboplatin/paclitaxel-based chemoradiotherapy in patients with stage III non-small cell lung cancer. Lung Cancer. 2021;159:56–65. doi: 10.1016/j.lungcan.2021.06.028 34311345

[31] Mehta MP, Wang D, Wang F, Kleinberg L, Brade A, Robins HI, et al. Veliparib in combination with whole brain radiation therapy in patients with brain metastases: results of a phase 1 study. Journal of Neuro-Oncology. 2015;122(2):409–17. doi: 10.1007/s11060-015-1733-1 25682091

[32] Appleman LJ, Beumer JH, Jiang Y, Lin Y, Ding F, Puhalla S, et al. Phase 1 study of veliparib (ABT-888), a poly (ADP-ribose) polymerase inhibitor, with carboplatin and paclitaxel in advanced solid malignancies. Cancer Chemotherapy and Pharmacology. 2019;84(6):1289–301. doi: 10.1007/s00280-019-03960-w 31549216 PMC7825275

[33] Reck M, Blais N, Juhasz E, Gorbunova V, Jones CM, Urban L, et al. Smoking History Predicts Sensitivity to PARP Inhibitor Veliparib in Patients with Advanced Non–Small Cell Lung Cancer. Journal of Thoracic Oncology. 2017;12(7):1098–108. doi: 10.1016/j.jtho.2017.04.010 28461256

[34] Chen AP, Kummar S, Moore N, Rubinstein LV, Zhao YD, Williams PM, et al. Molecular Profiling-Based Assignment of Cancer Therapy (NCI-MPACT): A Randomized Mulicentar Phase II Trial. Jco Precision Oncology. 2021;5:133–44. doi: 10.1200/po.20.00372 33928209 PMC8078898

[35] Lazzari C, Gregorc V, Bulotta A, Dottore A, Altavilla G, Santarpia M. Temozolomide in combination with either veliparib or placebo in patients with relapsed-sensitive or refractory small-cell lung cancer. Transl Lung Cancer Res. 2018;7:S329–s33. doi: 10.21037/tlcr.2018.12.02 30705847 PMC6328700

[36] McLouth LES, Zhao FM, Owonikoko TK, Feliciano JL, Mohindra NA, Dahlberg SE, et al. Patient-reported tolerability of veliparib combined with cisplatin and etoposide for treatment of extensive stage small cell lung cancer: Neurotoxicity and adherence data from the ECOG ACRIN cancer research group E2511 phase II randomized trial. Cancer Medicine. 2020;9(20):7511–23. doi: 10.1002/cam4.3416 32860331 PMC7571824

[37] Argiris A, Miao J, Cristea MC, Chen AM, s JM, Decker RH, et al. A Dose-finding Study Followed by a Phase II Randomized, Placebo-controlled Trial of Chemoradiotherapy With or Without Veliparib in Stage III Non–small-cell Lung Cancer: SWOG 1206 (8811). Clinical Lung Cancer. 2021;22(4):313–23.e1. doi: 10.1016/j.cllc.2021.02.009 33745865 PMC8562492

[38] Byers LA, Bentsion D, Gans S, Penkov K, Son C, Sibille A, et al. Veliparib in combination with carboplatin and etoposide in patients with treatment-Naïve extensive-stage small cell lung cancer: A phase 2 randomized study. Clinical Cancer Research. 2021;27(14):3884–95. doi: 10.1158/1078-0432.CCR-20-4259 33947690

[39] Owonikoko TK, Dahlberg SE, Sica GL, Wagner LI, Wade JL 3rd, Srkalovic G, et al. Randomized Phase II Trial of Cisplatin and Etoposide in Combination With Veliparib or Placebo for Extensive-Stage Small-Cell Lung Cancer: ECOG-ACRIN 2511 Study. J Clin Oncol. 2019;37(3):222–9. doi: 10.1200/JCO.18.00264 30523756 PMC6338394

[40] Pietanza MC, Waqar SN, Krug LM, Dowlati A, Hann CL, Chiappori A, et al. Randomized, Double-Blind, Phase II Study of Temozolomide in Combination With Either Veliparib or Placebo in Patients With Relapsed-Sensitive or Refractory Small-Cell Lung Cancer. J Clin Oncol. 2018;36(23):2386–94. doi: 10.1200/JCO.2018.77.7672 29906251 PMC6085179

[41] Ramalingam SS, Blais N, Mazieres J, Reck M, Jones CM, Juhasz E, et al. Randomized, Placebo-Controlled, Phase II Study of Veliparib in Combination with Carboplatin and Paclitaxel for Advanced/Metastatic Non-Small Cell Lung Cancer. Clin Cancer Res. 2017;23(8):1937–44. doi: 10.1158/1078-0432.CCR-15-3069 27803064

[42] Ramalingam SS, Novello S, Guclu SZ, Bentsion D, Zvirbule Z, Szilasi M, et al. Veliparib in Combination With Platinum-Based Chemotherapy for First-Line Treatment of Advanced Squamous Cell Lung Cancer: A Randomized, Multicenter Phase III Study. J Clin Oncol. 2021;39(32):3633–44. Epub 20210826. doi: 10.1200/JCO.20.03318 . 34436928 PMC8577684

[43] Govindan R, Lind M, Insa A, Khan SA, Uskov D, Tafreshi A, et al. Veliparib Plus Carboplatin and Paclitaxel Versus Investigator’s Choice of Standard Chemotherapy in Patients With Advanced Non–Squamous Non–Small Cell Lung Cancer. Clinical Lung Cancer. 2022;23(3):214–25. doi: 10.1016/j.cllc.2022.01.005 35331641

[44] Barayan R, Ran X, Lok BH. PARP inhibitors for small cell lung cancer and their potential for integration into current treatment approaches. Journal of Thoracic Disease. 2020;12(10):6240–52. doi: 10.21037/jtd.2020.03.89 33209463 PMC7656434

[45] Knelson EH, Patel SA, Sands JM. PARP Inhibitors in Small-Cell Lung Cancer: Rational Combinations to Improve Responses. Cancers [Internet]. 2021; 13(4). doi: 10.3390/cancers13040727 33578789 PMC7916546

[46] Bao Z, Cao C, Geng X, Tian B, Wu Y, Zhang C, et al. Effectiveness and safety of poly (ADP-ribose) polymerase inhibitors in cancer therapy: A systematic review and meta-analysis. Oncotarget. 2016;7(7):7629–39. Epub 2015/09/25. doi: 10.18632/oncotarget.5367 . 26399274 PMC4884943

[47] Schettini F, Giudici F, Bernocchi O, Sirico M, Corona SP, Giuliano M, et al. Poly (ADP-ribose) polymerase inhibitors in solid tumours: Systematic review and meta-analysis. European Journal of Cancer. 2021;149:134–52. doi: 10.1016/j.ejca.2021.02.035 33862496

[48] Jiang Y, Meng X-Y, Deng N-N, Meng C, Li L-H, He Z-K, et al. Effect and Safety of Therapeutic Regimens for Patients With Germline BRCA Mutation-Associated Breast Cancer: A Network Meta-Analysis. Frontiers in oncology. 2021;11. doi: 10.3389/fonc.2021.718761 34490117 PMC8417748

[49] Ratta R, Guida A, Scotté F, Neuzillet Y, Teillet AB, Lebret T, et al. PARP inhibitors as a new therapeutic option in metastatic prostate cancer: a systematic review. Prostate Cancer and Prostatic Diseases. 2020;23(4):549–60. doi: 10.1038/s41391-020-0233-3 32367009

[50] Jiang Y, Zhao J, Zhang L, Tian S, Yang T, Wang L, et al. Evaluation of the Efficacy and Safety of PARP Inhibitors in Advanced-Stage Epithelial Ovarian Cancer. Frontiers in oncology. 2020;10. doi: 10.3389/fonc.2020.00954 32719741 PMC7350528

[51] Hao J, Liu Y, Zhang T, He J, Zhao H, An R, et al. Efficacy and safety of PARP inhibitors in the treatment of advanced ovarian cancer: An updated systematic review and meta-analysis of randomized controlled trials. Critical Reviews in Oncology/Hematology. 2021;157:103145. doi: 10.1016/j.critrevonc.2020.103145 33254040

[52] Chang X-F, Ren X-L, Yang J-Q, Shi J-J, Bai J-H, Cui M-S, et al. Evaluation of efficacy and safety of PARP inhibitors in breast cancer: A systematic review and meta-analysis. The Breast. 2021;59:44–50. doi: 10.1016/j.breast.2021.05.009 34130011 PMC8215282

[53] Stemmer A, Shafran I, Stemmer SM, Tsoref D. Comparison of Poly (ADP-ribose) Polymerase Inhibitors (PARPis) as Maintenance Therapy for Platinum-Sensitive Ovarian Cancer: Systematic Review and Network Meta-Analysis. Cancers [Internet]. 2020; 12(10). doi: 10.3390/cancers12103026 33081005 PMC7603267

