# Supplementary material for: Efficacy and safety of veliparib plus chemotherapy for the treatment of lung cancer: A systematic review of clinical trials
Source: PLoS One. 2023 Sep 8;18(9):e0291044. doi: 10.1371/journal.pone.0291044 (PMC10490931; doi:10.1371/journal.pone.0291044)
Supplement: S1 Table — (DOCX) [file pone.0291044.s001.docx]

**S1 Table.** Search strategy for PubMed, Scopus, Web of Science and Google Scholar.

| **Database**  **(search date)** | **Step** | **Search strategy** | **Number of results** |
| --- | --- | --- | --- |
| PubMed  (10.30.2022) | #1 | “Veliparib”[Supplementary Concept] OR “Veliparib”[tiab] OR “ABT-888”[tiab] OR “ABT888”[tiab] OR “ABT 888”[tiab] OR “NSC 737664”[tiab] | 538 |
|  | #2 | “Lung Neoplasms”[mh] OR “Pulmonary Blastoma”[mh] OR ((“Lung”[mh] OR “Lung*”[tiab] OR “Pulmonary”[tiab]) AND (“Neoplasms”[mh] OR “Neoplas*”[tiab] OR “Cancer*”[tiab] OR “Tumor*”[tiab] OR “Tumour*”[tiab] OR “Malignan*”[tiab] OR “Carcinoma*”[tiab] OR “Carcinoid*” OR “Adenocarcinoma*”[tiab] OR “Lymphoma*”[tiab] OR “Sarcoma*”[tiab] OR “blastoma*”[tiab] OR “Fibrosarcoma*”[tiab] OR “Leiomyosarcoma*”[tiab])) | 474,529 |
|  | #3 | #1 AND #2 | 56 |
| Scopus  (10.30.2022) | #1 | TITLE-ABS-KEY ( “Veliparib” OR “ABT-888” OR “ABT888” OR “ABT 888” OR “NSC 737664” ) | 1,985 |
|  | #2 | TITLE-ABS-KEY ( (“Lung*” OR “Pulmonary”) AND (“Neoplas*” OR “Cancer*” OR “Tumor*” OR “Tumour*” OR “Malignan*” OR “Carcinoma*” OR “Carcinoid*” OR “Adenocarcinoma*” OR “Lymphoma*” OR “Sarcoma*” OR “blastoma*” OR “Fibrosarcoma*” OR “Leiomyosarcoma*”) ) | 694,415 |
|  | #3 | #1 AND #2 | 383 |
| Web of Science  (10.30.2022) | #1 | TS=(“Veliparib” OR “ABT-888” OR “ABT888” OR “ABT 888” OR “NSC 737664”) | 915 |
|  | #2 | TS=((“Lung*” OR “Pulmonary”) AND (“Neoplas*” OR “Cancer*” OR “Tumor*” OR “Tumour*” OR “Malignan*” OR “Carcinoma*” OR “Carcinoid*” OR “Adenocarcinoma*” OR “Lymphoma*” OR “Sarcoma*” OR “blastoma*” OR “Fibrosarcoma*” OR “Leiomyosarcoma*”)) | 514,275 |
|  | #3 | #1 AND #2 | 98 |
| Google Scholar  (11.5.2022) |  | (“Veliparib” OR “ABT-888”) AND (“Lung Cancer*” OR “Pulmonary Cancer*”) | 4,980 |
