# Supplementary material for: Efficacy and safety of veliparib plus chemotherapy for the treatment of lung cancer: A systematic review of clinical trials
Source: PLoS One. 2023 Sep 8;18(9):e0291044. doi: 10.1371/journal.pone.0291044 (PMC10490931; doi:10.1371/journal.pone.0291044)
Supplement: S2 Table — (DOCX) [file pone.0291044.s002.docx]

**S2 Table.** Quality assessment of the included studies.

| **Study ID** | **D1. Bias arising**  **from the**  **randomization**  **process** | **D2. Bias due to**  **deviations from**  **the intended**  **interventions** | **D3. Bias due to missing**  **outcome data** | **D4. Bias in the**  **measurement**  **of the outcome** | **D5. Bias in the**  **selection of the**  **reported result** | **Overall bias** |
| --- | --- | --- | --- | --- | --- | --- |
| Argiris et al. 2021 (1) | Some Concerns | Some Concerns | Low Risk | High Risk | Low Risk | High Risk |
| Byers et al. 2021 (2) | Some Concerns | Some Concerns | Low Risk | High Risk | Low Risk | High Risk |
| Govindan et al. 2022 (3) | Low Risk | Some Concerns | Low Risk | High Risk | High Risk | High Risk |
| Owonikoko et al. 2019 (4) | Some Concerns | Some Concerns | Low Risk | High Risk | Low Risk | High Risk |
| Pietanza et al. 2018 (5) | Some Concerns | Some Concerns | Low Risk | High Risk | Low Risk | High Risk |
| Ramalingam et al. 2017 (6) | Some Concerns | Some Concerns | Low Risk | High Risk | Low Risk | High Risk |
| Ramalingam et al. 2021 (7) | Some Concerns | Some Concerns | Low Risk | High Risk | Low Risk | High Risk |

RoB2 overall risk of bias judgment

Low risk of bias → The study is judged to be at low risk of bias for all domains for this result.

Some concerns → The study is judged to raise some concerns in at least one domain for this result, but not to be at high risk of bias for any domain.

High risk of bias → The study is judged to be at high risk of bias in at least one domain, or to have some concerns for multiple domains in a way that substantially lowers confidence in the result.

**References**

1. Argiris A, Miao J, Cristea MC, Chen AM, s JM, Decker RH, et al. A Dose-finding Study Followed by a Phase II Randomized, Placebo-controlled Trial of Chemoradiotherapy With or Without Veliparib in Stage III Non–small-cell Lung Cancer: SWOG 1206 (8811). Clinical Lung Cancer. 2021;22(4):313-23.e1.

2. Byers LA, Bentsion D, Gans S, Penkov K, Son C, Sibille A, et al. Veliparib in combination with carboplatin and etoposide in patients with treatment-Naïve extensive-stage small cell lung cancer: A phase 2 randomized study. Clinical Cancer Research. 2021;27(14):3884-95.

3. Govindan R, Lind M, Insa A, Khan SA, Uskov D, Tafreshi A, et al. Veliparib Plus Carboplatin and Paclitaxel Versus Investigator's Choice of Standard Chemotherapy in Patients With Advanced Non–Squamous Non–Small Cell Lung Cancer. Clinical Lung Cancer. 2022;23(3):214-25.

4. Owonikoko TK, Dahlberg SE, Sica GL, Wagner LI, Wade JL, 3rd, Srkalovic G, et al. Randomized Phase II Trial of Cisplatin and Etoposide in Combination With Veliparib or Placebo for Extensive-Stage Small-Cell Lung Cancer: ECOG-ACRIN 2511 Study. J Clin Oncol. 2019;37(3):222-9.

5. Pietanza MC, Waqar SN, Krug LM, Dowlati A, Hann CL, Chiappori A, et al. Randomized, Double-Blind, Phase II Study of Temozolomide in Combination With Either Veliparib or Placebo in Patients With Relapsed-Sensitive or Refractory Small-Cell Lung Cancer. J Clin Oncol. 2018;36(23):2386-94.

6. Ramalingam SS, Blais N, Mazieres J, Reck M, Jones CM, Juhasz E, et al. Randomized, Placebo-Controlled, Phase II Study of Veliparib in Combination with Carboplatin and Paclitaxel for Advanced/Metastatic Non-Small Cell Lung Cancer. Clin Cancer Res. 2017;23(8):1937-44.

7. Ramalingam SS, Novello S, Guclu SZ, Bentsion D, Zvirbule Z, Szilasi M, et al. Veliparib in Combination With Platinum-Based Chemotherapy for First-Line Treatment of Advanced Squamous Cell Lung Cancer: A Randomized, Multicenter Phase III Study. J Clin Oncol. 2021;39(32):3633-44.
